# Cost-effectiveness of seasonal malaria chemoprevention in upper west region of Ghana

**DOI:** 10.1186/s12936-016-1418-z

**Published:** 2016-07-16

**Authors:** Justice Nonvignon, Genevieve Cecilia Aryeetey, Shamwill Issah, Patrick Ansah, Keziah L. Malm, Winfred Ofosu, Titus Tagoe, Samuel Agyei Agyemang, Moses Aikins

**Affiliations:** Department of Health Policy, Planning and Management, School of Public Health, College of Health Sciences, University of Ghana, P. O. Box LG13, Legon, Ghana; UK Department for International Development, Accra, Ghana; Navrongo Health Research Centre, Ghana Health Service, Navrongo, Ghana; National Malaria Control Programme, Ghana Health Service, Accra, Ghana; Upper West Regional Health Directorate, Ghana Health Service, Wa, Ghana

**Keywords:** Seasonal malaria chemoprevention, Incremental cost-effectiveness ratios, Under-five, Ghana

## Abstract

**Background:**

In Ghana, malaria is endemic and perennial (with significant seasonal variations in the three Northern Regions), accounting for 33 % of all deaths among children under 5 years old, with prevalence rates in children under-five ranging from 11 % in Greater Accra to 40 % in Northern Region. Ghana adopted the WHO-recommended Seasonal Malaria Chemoprevention (SMC) strategy with a trial in the Upper West Region in 2015. The objective of this study was to estimate the cost-effectiveness of seasonal malaria chemoprevention.

**Methods:**

Costs were analysed from provider and societal perspectives and are reported in 2015 US$. Data on resource use (direct and indirect costs) of the SMC intervention were collected from intervention records and a survey in all districts and at regional level. Additional numbers of malaria cases and deaths averted by the intervention were estimated based on prevalence data obtained from an SMC effectiveness study in the region. Incremental cost-effectiveness ratios (ICERs) were estimated for the districts and region. Sensitivity analyses were conducted to test the robustness of the ICERs.

**Results:**

The total financial cost of the intervention was US$1,142,040.80. The total economic cost was estimated to be US$7.96 million and US$2.66 million from the societal and provider perspectives, respectively. The additional numbers of cases estimated to be averted by the intervention were 24,881 and 808, respectively. The economic cost per child dosed was US$67.35 from societal perspective and US$22.53 from the provider perspective. The economic cost per additional case averted was US$107.06 from the provider perspective and US$319.96 from the societal perspective. The economic cost per additional child death averted by the intervention was US$3298.36 from the provider perspective and US$9858.02 from the societal perspective. The financial cost per the SMC intervention delivered to a child under-five was US$9.66. The ICERs were sensitive to mortality rate used.

**Conclusions:**

The SMC intervention is economically beneficial in reducing morbidity in children under-5 years and presents a viable approach to improving under-five health in Ghana.

## Background

The global burden of malaria reduced by about 18 % at 2015 (from 262 million in 2000 to 214 million in 2015) according to recent World Health Organization (WHO) estimates [1]. During the same period, malaria incidence, mortality rate and deaths were estimated to have fallen by 37, 60 and 48 %, respectively. In spite of the major achievements in the fight against malaria, the disease continues to pose a major public health challenge, especially in sub-Saharan Africa (SSA), which accounted for about 88 % of the global cases and 90 % of deaths in 2015 [1]. Deaths in children under-5 years old in SSA reduced by about 57.9 %, moving malaria from the leading to the fourth cause of death in under-fives in Africa. Thus, malaria accounted for about 10 % of under-five deaths in SSA in 2015 [1].

In Ghana, malaria has consistently remained the leading cause of morbidity and mortality accounting for about 38 % of all outpatient visits and 27.3 % of all admissions and responsible for 48.5 % of all deaths among children below age 5 years in 2015 [2]. Malaria prevalence rates in children under-five ranges from 11 % in Greater Accra to 40 % in Northern Region [3]. Ghana was one of the ten African countries that accounted for more than 60 % of malaria deaths in SSA in 2012 [4].

Despite the many interventions in place, it appears the burden of malaria remains high and new preventive/control measures are of necessity to augment the existing measures. Seasonal malaria chemoprevention (SMC), formerly known as intermittent preventive treatment in children (IPTc), has been identified as an effective strategy in areas with a short malaria transmission season. For example, in a systematic review/meta-analysis of SMC studies, Wilson [5] showed that giving children under-5 years of age sulfadoxine–pyrimethamine plus amodiaquine (SP-AQ) once per month during the peak malaria transmission season reduced the incidence of clinical attacks of malaria by 83 % and severe malaria by 77 %. Further, a Cochrane review by Meremikwu et al. [6] reported the SP-AQ combination therapy as safe. SMC is a community-level intervention and the use of community health workers for implementation has proven to increase coverage and cost effectiveness [7, 8]. The administration of SMC medicines is usually done monthly for 3–4 months during the peak malaria transmission season.

Against this background, the WHO in 2011 [9] recommended incorporation of SMC into malaria control programmes in areas with highly seasonal transmission of malaria. By 2014, six countries in SSA (Chad, The Gambia, Guinea, Mali, Niger, and Senegal), had adopted the SMC strategy, with two others (Togo and Congo) reporting policy to adopt it. Ghana started implementation in 2015.

Ghana can be stratified roughly into three malaria epidemiologic zones: northern savannah, tropical rainforest, and coastal savannah/mangrove swamps. The length of malaria transmission season varies by geographic region. There are two major transmission patterns. In the northern part of the country, there is a 6- to 7-month transmission season, with the highest number (50–60 %) of cases occurring between July and November. In the southern part of the country, malaria is endemic throughout the year with some heightened transmission during May to June and a larger peak in October to November. Malaria prevalence and seasonality makes Ghana, and its northern regions in particular, an excellent choice for implementing the SMC intervention [10]. Ghana piloted SMC intervention in July to October 2015, with a trial in the Upper West Region (which has one of the highest prevalence of malaria in the country—37.8 %) through support from the Department for International Development of the UK (DFID) and the Global Fund. Lessons learnt from this implementation were to be used in scaling up SMC in other eligible northern regions of Ghana. The intervention used the services of volunteers supervised by district and sub-district health workers.

Studies have shown that different delivery models of various malaria control strategies are cost-effective (i.e., represent a good use of society’s resources). For instance, insecticide-treated net (ITN) campaigns are cost-effective in reducing morbidity and mortality in children in Ghana [11, 12], Uganda [13], Tanzania [14–16], and Togo [17]. Further, indoor residual spraying (IRS) has been shown to be a cost-effective malaria vector control strategy in Mozambique [18, 19], Kenya [20], South Africa [19], and SSA [21]. Conteh et al. [22], Conteh et al. [23] and Ross et al. [8] present the cost-effectiveness of different strategies for delivering intermittent preventive treatments in children and infants. Again, there is evidence that malaria diagnostic and treatment strategies present value-for-money to society. These studies and others have shown that the WHO-recommended approach of using trained community health volunteers to diagnose and treat uncomplicated fevers (including malaria) in children near their home is cost-effective (Ghana [24]; Uganda [25]; Zambia [26]).

Although IPTc has been shown to be cost-effective elsewhere [8, 22] and in Ghana [23], there is paucity of studies assessing the cost-effectiveness of SMC in its current approach (and using the combination of anti-malarials) as recommended by the WHO in 2012. The focus of the study by Conteh et al. [23] was on delivery strategies of IPTc using SP or AS (Artesunate)–AQ, whereas SMC uses SP plus AQ. Thus, it is important to assess the cost-effectiveness of SMC in the Ghanaian context to inform scale-up. The findings of this study will be useful for Ghana and other SSA countries that are in the process of scaling-up or implementing SMC as recommended by WHO.

The aim of this study was to estimate the cost-effectiveness of SMC intervention delivered to children under-5 years old in the Upper West Region of Ghana.

## Methods

### SMC intervention

**Fig. 1:**
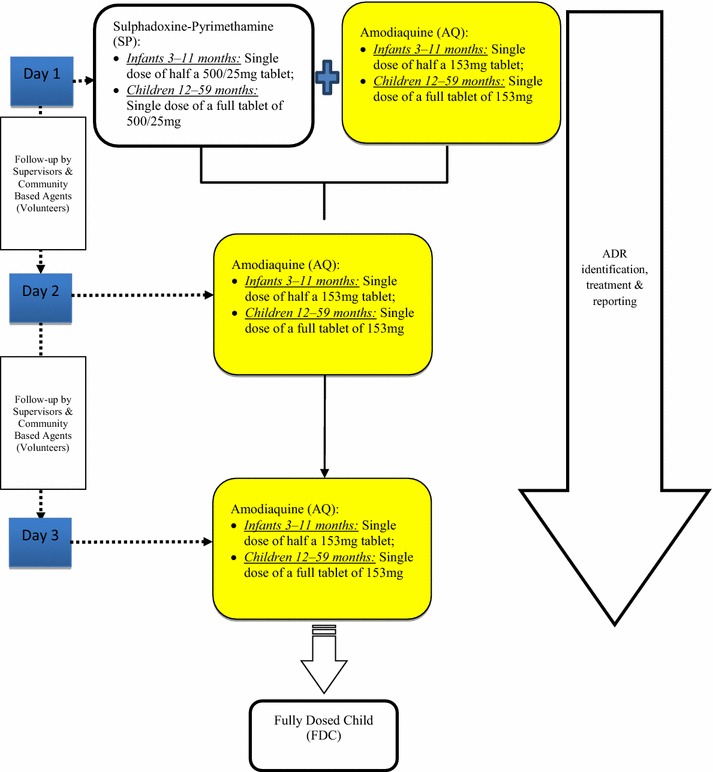
Algorithm for seasonal malaria chemoprevention (SMC)

Following WHO recommendations on administration of SMC to children under-5 years, all eligible children were given full treatment doses of sulfadoxine–pyrimethamine (SP) and amodiaquine (AQ) at monthly intervals from mid-June to mid-September 2015. Each child was given four courses of the treatment (i.e., a fully dosed child). Dosing schedules are shown in Fig. 1.

The recommended anti-malarials (AQ-SP) were administered to each child as directly observed therapy as scheduled in each cycle. There were 3 days for dosing children and 2 days for follow-up on pharmaco-vigilance per cycle. Volunteers visited each household and the target children were given the required number of doses of the anti-malarial as scheduled. An active pharmaco-vigilance system was established to capture adverse drug reactions (ADR) in children who received SMC in each intervention district. This was done following the guideline outlined under reporting of adverse events (i.e., mild, moderate, severe, and life-threatening). The intervention was carried out in four rounds in all 11 districts: Round 1 was from 20–29 July; Round 2 was from 24–29 August; Round 3 was from 5–10 October; and, Round 4 was from 9–14 November, 2015.

### Study setting

The study was conducted in the Upper West Region of Ghana, located in northwestern Ghana. It is bordered to the south by the Northern Region, to the north and west by Burkina Faso and to the east by the Upper East region. With an area of 18,476 sq km, the region’s population density stands at 40 persons per sq km. The projected population for 2015 based on the 2010 Population and Housing Census growth rate of 1.9 % is 771,394. The climate is tropical with an average minimum temperature of 22.6 °C and maximum of 40.0 °C. There are two main seasons: the rainy season begins around April and ends in October with peaks in July, August and September. The dry season commences in late November to the end of April. The dry season is characterized by harsh climatic conditions of dryness, high temperatures and dust. The Region has been divided into 11 administrative districts, which correspond to the division of the health sector.

The Regional Health Management Team (RHMT) oversees planning and implementation of health services in the Region. There are 11 district health management teams (DHMT) with 66 sub-districts. There is a total of 249 health facilities in the region: one regional hospital, three district government hospitals, three private hospitals and two mission hospitals, five policlinics, 65 health centres, 14 clinics, five maternity homes, and 194 community-based health planning and services (CHPS) compounds. Health services such as clinical care, reproductive and child health, nutrition, immunization, and other disease control services are delivered in all the districts with support from a community-based health system of volunteers and service providers, made up of 1209 traditional birth attendants, 1850 community-based surveillance volunteers, 3520 community-based agents, and 184 guinea worm volunteers who are providing various services in 3557 demarcated areas, with supervision from sub-district health staff. There are also 861 outreach points for the provision of immunization and other services.

### Study population and sampling

The study population for the SMC intervention was children between age 3 and 59 months in all 11 districts. Given the nature of the study, which involved use of community volunteers and participation of caregivers, the sampling procedure involved selection of volunteers and caregivers from all 11 districts.

In each district, two sub-districts were randomly selected. Sub-district supervisors, mainly health workers in the sub-district, then identified the volunteers they supervised, and these volunteers were interviewed. Subsequently, each volunteer interviewed led the research team to interview caregivers whose children were dosed by the volunteers. The caregivers were also interviewed. In all, 112 volunteers and 517 caregivers were interviewed.

### SMC effectiveness study

The intervention was implemented in all 11 districts of the Upper West Region. However, an effectiveness study was undertaken to ascertain the impact of SMC on malaria morbidity and mortality in children under-5 years using a cluster-randomized comparative study design with one district randomly selected in the intervention area and another in the Northern Region (a control region) where SMC was not implemented. The Northern Region is similar to the Upper West Region in terms of malaria transmission and other social infrastructure, such as the health service delivery system. Most childhood mortality and morbidity from malaria occurs during the rainy season. The intervention district selected was Lawra District (upper west region) and the control district West Mamprusi District (northern region). The estimated prevalence in this case–control study was used to estimate the caseload with and without SMC in the study area.

### Data collection

Both financial and economic costs were measured. Financial costs were actual expenditure incurred in undertaking the SMC intervention, whilst economic costs were broader and dealt with opportunity cost (alternative use) of using the resources in the intervention (i.e., direct and indirect costs to both volunteers and caregivers were incorporated). Estimated financial costs were useful for developing the budget for scale-up and the economic costs were used for estimating the cost-effectiveness of the intervention. An ingredient approach was used to identify all resources required to implement the SMC dosing programme throughout the region. Financial costs were obtained retrospectively from the SMC programme reports and accounts of the region and district in the custody of the malaria focal persons and accounting officers. Research and evaluation costs were not included. Costs were measured in Ghanaian Cedis (GHS) and pound sterling (GBP), depending on the currency of the original expenditure. Replacement costs of all capital items, such as vehicles, were obtained from the open market. Costs in GHS and GBP were converted to US$ according to Bank of Ghana’s average annual exchange rate for the year of the expenditure (US$1.00 equivalent to GHS3.74 in 2015).

Economic costs refer to opportunity costs and include costs of donated goods, such as AMOFAN medication or volunteered time spent on the intervention by volunteers and caregivers. Data on time spent by volunteers and caregivers as part of the SMC intervention were obtained from two inter-related surveys. A cross-sectional survey of volunteers and caregivers participating in the SMC dosing exercises was undertaken purposively to determine their productivity losses. The data collection tool of volunteers covered: (1) demographics; and, (2) productivity loss (information on the time that volunteers spent on SMC training and dosing exercises, what they would have been doing if not working on SMC and the local farm wage rate for a day. Whilst the caregiver instrument was made up of: (1) demographics; (2) self-medication for malaria treatment; (3) insect bite preventive measures; (4) other household direct costs; and, (5) other household indirect costs (i.e., information on the time that caregivers spent on SMC dosing exercises and the amount of time caregivers had to wait at home for the dosing visit, what they would have been doing if not wait and engaging in the SMC dosing).

Field data collection was carried out from 10–27 November, 2015 in all 11 districts of the region. This was also the schedule period for the fourth round of dosing. Both volunteers and caregivers were verbally consented before the administration of the questionnaire. With the assistance of the district malaria focal-persons, four or more volunteers per district were purposely selected for the interview depending on their availability, since the data collection period coincided with the fourth-round dosing and their farming season. Most volunteers and caregivers are primarily farmers. After the interview with each volunteer, using the snowball approach, four or more caregivers who had dosed their children (3–59 months) were identified, consented and interviewed. A total of 122 volunteers and 512 caregivers were interviewed in all 11 districts. Additionally, the research team used a checklist to observe and time-track some volunteers during the dosing exercise.

The Regional Health Administration (RHA) facilitated data collection in all 11 districts. The malaria focal-persons in the 11 districts were interviewed on the resources they used in all four rounds of the implementation of SMC. The interview covered the following activities: (1) planning; (2) social mobilization; (3) training of health workers; (4) training of volunteers (community-based agents); (5) dosing exercise; (6) monitoring and supervision; (7) data capture; (8) pharmaco-vigilance; and, (9) post-SMC feedback/review meeting. In all these activities, the resources used in terms of recurrent and capital items were identified and enumerated. The available costs of these items were then collected from the malaria focal persons and the District Accounts Officer. For most of the capital items, their quantities and brand/type were ascertained and their replacement costs were obtained from the open market.

### Data analysis

The data analyses covered cost analysis and estimation of the effectiveness of SMC dosing.

### Cost analysis

Costing was undertaken from both provider perspective (which included provider-related costs incurred only on delivery of the intervention) and societal perspective (which included cost incurred on delivery of the intervention, donations and the time and other expenses of caregivers). The analytic horizon was 4 months, which constituted the duration of the SMC implementation.

Financial and economic costs were analysed separately. Two categories of economic costs were considered in the analysis: direct costs and indirect costs of all intervention activities. The direct costs were direct expenses incurred on programme activities and on capital and recurrent components. The capital costs included costs incurred on items with useful lives of more than 1 year, including vehicles, equipment, etc. The cost information was based on the entire duration of project implementation, from the planning phase to the end of the intervention phase. All capital goods with an expected lifespan of more than 1 year were annualized using a discount rate of 3 % according to the guidelines of standard methods [24]. The lifespan of vehicles, such as pick-up trucks and motorbikes used in SMC activities, was estimated to be 5 and 3 years, respectively, based on information received from the RHA Transport Manger. An average useful lifespan of 3 years was assumed for office equipment, such as computer and printers. Other regional-level costs associated with SMC, such as planning, stakeholders meetings, monitoring and supervision, and post-SMC programme meetings were apportioned to the districts according to the number of sub-districts.

Recurrent costs included cost of items with useful lives of less than 1 year (e.g. medicine supplies, stationery supplies, salaries and allowances of staff, etc.). The time of project personnel, health workers, volunteers and other staff involved in training were valued, using their gross monthly salaries, and added to the recurrent cost of the respective activity. The time of staff of Ghana Health Service and other stakeholders, who were directly compensated for participation in the intervention, was valued based on the standard Ghanaian Single Spine Salary structure as an economic cost. These allowances were based on a pre-determined market premium of each category of staff. The salary grades of all health workers involved in the SMC intervention were obtained from the Regional Administrator. The numbers of days worked on the SMC intervention were obtained from the various districts’ malaria focal persons.

Data on indirect costs covered both volunteers’ and caregivers’ time and productivity losses (i.e., work days lost by engaging in SMC dosing). The total number of volunteers involved in the SMC programme was ascertained for each district. The monetary value of work days lost was calculated by multiplying number of work days lost with the prevailing local agriculture wage rate (a theoretical measure of labour productivity) in the district. Summation of capital cost and recurrent cost constituted total financial or economic cost.

### Measurement of SMC effectiveness

The number of children under-5 years dosed during the programme was obtained from the Regional Malaria Control Programme office, Upper West Regional Health Directorate for all four rounds of the intervention. The coverage for each district was used to estimate the number of fully dosed children under-5 years for each district. The measures of effect were:

#### Additional number of cases averted

Based on the prevalence of 31 % obtained from the SMC effectiveness study conducted by the Navrongo Health Research Centre, incidence of malaria during the transmission season (when SMC was given) was estimated as 350 per 1000 children, following the approach described in Cairns et al. [27]. As described by Cairns et al. [27], this is likely to be a conservative estimate of the incidence rate in young children. The total case-load during the SMC period in Upper West region was estimated by applying this incidence rate to the total population of children under-5 years. Based on administrative coverage of around 80, a conservative assumption of population coverage of SMC of 60 % and efficacy of SMC in those who receive it was 80 % [27] were made. The burden during the season, *b*, was therefore reduced by adding together the (unaffected) burden in the 40 % who did not receive SMC, with the reduced burden in those who received SMC:

$$b * 0.4 + b * 0.6 * \left( {1 - 0.8} \right) = b * 0.52$$

This translates into a 48 % reduction in the estimated burden.

#### Additional number of all-cause under-five deaths averted

Mortality rate of 11.4 deaths per 1000 under-five population for rural high transmission areas (estimated by Rowe et al. [28] and used in World Malaria Report 2008) was used to derive malaria deaths in the population. Then the SMC efficacy and administrative coverage were applied to the estimated deaths in the population to derive the number of deaths with SMC intervention. The difference between the estimated deaths with and without SMC constituted the number of deaths averted by SMC.

### Estimating the incremental cost-effectiveness of SMC

The SMC intervention was considered as an addition to existing health service. Thus, incremental cost-effectiveness ratios (ICER) of SMC were calculated as the incremental costs divided by the incremental effects, i.e., the additional effect (cases averted or deaths averted). A Microsoft Excel template was developed and used in calculating costs and cost-effectiveness ratios.

### Sensitivity analysis

Sensitivity analyses of ICERs were undertaken using varying key cost and effectiveness indicators, which had some degree of uncertainty. Key cost indicators varied, including medicine cost, discount rate, wage rate, protective efficacy, and deaths averted. Each was varied in turn in one-way sensitivity analyses. First, the cost of AMOFAN (SP/AQ) was inflated using the 2015 annual health consumer price index of 16.1 %. Second, a discount rate of 5 % was used as the mid-value of the lower and upper values of 0 and 10 % [29]. Third, the national minimum wage rate of GHS7.00 per day was used to value all volunteer time in place of the local agricultural wage rate. Fourth, Tagbor et al. [30] estimated protective efficacy of 38.5 % was used in place of the pooled estimate of 80 % efficacy. Again, actual administrative SMC coverage of 80 % was used in place of the conservative coverage of 60 % used in the analysis. Finally, to explore the importance of assumptions regarding estimated deaths averted using SMC, the constant mortality rate of 4.5 deaths per 1000 population and ten deaths per 1000 population [27] were used separately in the sensitivity analysis.

## Results

A total of 148,104 children under-5 years of age in the Upper West Region were estimated as eligible for dosing using regional census data. Out of this figure, 79.8 % received complete doses (i.e., four rounds).

### Costs of SMC intervention

#### Financial costs

**Fig. 2:**
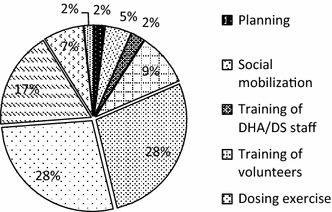
Financial cost profile by activity (%)

The total financial cost of the intervention in the region was US$1142,040.80. The minimum financial cost was US$61,571.06 in Dafiamma-Bussie-Issa (DBI) district and the maximum was US$253,308.45 in Sissala West district (Table 1). Recurrent cost constituted 95 % of the total financial cost. The dosing exercise and monitoring and supervision accounted for 27.7 and 27.6 %, respectively, of the total financial cost. Data capture, training (staff and volunteer) and pharmaco-vigilance accounted for 17.3, 11.9 and 7.2 %, respectively (Fig. 2).

**Table 1:** Cost-effectiveness of smc programme in the upper west region

Item	Region
Target population (children under 5 years)	148,104.00
Number of fully dosed children	118,208.00
Cost of SMC programme (US$, 2015 prices)	
Total financial cost	1142,040.80
Financial cost per fully dosed child	9.66 (95 % CI 7.46–14.21)
Economic cost (provider perspective)	2663,697.18
Economic cost (societal perspective)	7961,153.27
Effect of SMC programme	
Additional number of cases averted	24,881
Additional number of child deaths averted	808
Cost-effectiveness ratios (ICERs) (US$, 2015 prices)	
Provider perspective^a^	
Economic cost per fully dosed child	22.53 (95 % CI 21.08–28.06)
Economic cost per additional case averted	107.06 (95 % CI 99.75–121.48)
Economic cost per additional child death averted	3298.36 (95 % CI 3073.26–3742.64)
Societal perspective^b^	
Economic cost per fully dosed child	67.35 (95 % CI 63.56–77.86)
Economic cost per additional case averted	319.96 (95 % CI 284.23–366.38)
Economic cost per additional child death averted	9858.02 (95 % CI 8757.11–11,288.08)

^a^Includes only provider-related costs incurred on delivery of the intervention

^b^Includes cost incurred on delivery of the intervention, donations and the valued time and other expenses of caregivers and volunteers

#### Financial cost per fully dosed child

**Fig. 3:**
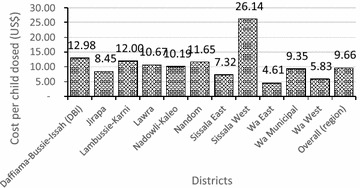
Financial cost per fully dosed child per district

**Fig. 4:**
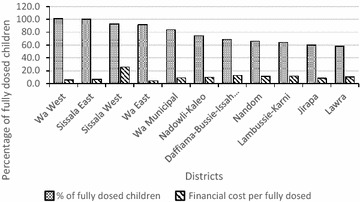
Percentage of fully dosed child vs. financial cost per district

The (average) financial cost per fully dosed child (four rounds of SMC) for the Region was US$9.66 (95 % CI 7.46–14.21), ranging from US$4.61 in Wa East to US$26.14 in Sissala West (Fig. 3). Figure 4 shows the economies of scale of the dosing exercise where a higher percentage of fully dosed children leads to lower financial cost per fully dosed child, with the exception of Sissala West.

### Economic costs of SMC

**Fig. 5:**
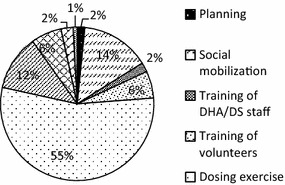
Economic cost profile by activity (%)

The total economic cost of the SMC intervention in the Region was estimated to be approximately US$7.96 million from the societal perspective and US$2.66 million from the provider perspective (Table 1). For the societal costs, DBI recorded the least cost of US$389,192.38, whilst Wa Municipal recorded the highest economic cost of US$1175,496.28. For the provider costs, DBI recorded the least cost of US$133,891.17 whilst Wa East recorded the highest economic cost of US$347,268.45. Indirect costs accounted for about 74 % of the total societal costs and 24 % of the total provider costs. Recurrent cost constituted 99 % of the total societal economic cost. Figure 5 shows that by activity, the dosing exercise accounted for about 55 % of the total societal economic cost, followed by social mobilization (about 15 %) and monitoring and supervision (about 12 %). Training (of staff and volunteers) accounted for about 8 % of the total economic cost.

### Effects of SMC intervention

Table 1 shows that the additional number of under-five malaria cases averted by the intervention in the Region was 24,881, representing 48 % of the total caseload without SMC. The lowest number of additional cases averted was recorded in DBI (1155) and the highest was recorded in Wa East (3799). Additionally, Table 2 shows that a total number of 808 deaths was estimated to be averted by the intervention. The estimated lowest and highest child deaths averted were recorded in DBI (37) and Wa East (123) respectively.

**Table 2:** Sensitivity analysis results

Parameter	Change	ICERs (provider perspective)		ICERs (societal perspective)	
		Cost per additional case averted	Cost per additional death averted	Cost per additional case averted	Cost per additional death averted
Base estimates	Base case scenario	107.06	3298.38	319.96	9858.02
Medicine cost	Application of 16.1 % inflation rate	112.55	3467.77	325.46	10,027.43
Discount rate	Increasing the discount rate from 3 to 5 %	107.24	3304.16	320.15	9863.81
National min. wage rate	Use of national daily minimum wage rate of GHS7 rather than agric wage of GHS 11.36	97.33	2328.26	228.48	6958.60
Mortality rate	10 deaths per 1000 population	107.60	2998.65	319.96	7039.40
Mortality rate	4.5 deaths per 1000 population	107.60	6853.74	319.96	20,484.19
SMC coverage	Increase in SMC administrative coverage from 60 to 80 %	107.60	10,654.79	319.96	31,996.31
SMC protective efficacy	Decrease in protective efficacy from 80 to 38.5 %	222.45	23,997.27	664.86	71,102.91

### Incremental cost effectiveness ratios (ICERs)

Table 1 shows that at the regional level, the economic cost per child dosed from the provider perspective was US$22.53 (95 % CI 21.08–28.06), with Wa West recording the lowest (US$15.39) and Lambussie-Karni recording the highest (US$33.26), respectively. From the societal perspective, the economic cost per child dosed was US$67.35 (95 % CI 63.56–77.86), ranging from US$46.37 in Wa East to US$92.74 in Wa Municipal. Table 1 further shows that the economic cost per additional case averted by the intervention from the provider perspective was US$107.06 (95 % CI 99.75–121.48), ranging from US$83.25 in Jirapa to US$135.31 in Sissala West. From the societal perspective, the economic cost per additional case averted was US$319.96 (95 % CI: 284.23–366.38), with the lowest recorded by Lawra (US$244.39) and highest by Wa Municipal (US$460.24).

The results show that the economic cost per additional child death averted by the intervention was US$3298.36 (95 % CI 3073.26–3742.64), from the provider perspective and US$9858.02 (95 % CI 8757.11–11,288.08), from the societal perspective. For the former, the lowest ICER was recorded by Jirapa (US$2655.04) and highest by Sissala West (US$4168.78). From the societal perspective, the range of ICERs was US$14,179.87 and US$7529.68 recorded by Wa Municipal and Lawra, respectively (Table 1).

### Sensitivity analysis

The ICERs were sensitive to the mortality rates used in estimating deaths averted. For instance, the use of ten deaths per 1000 population [27] increased the economic cost per additional death averted by about 2.2 times. Similarly, the use of 4.5 deaths per 1000 population [27] increased the economic cost per additional death averted by about 6.3 times The ICERs were least sensitive to the discount rate, minimum wage and inflation in medicine cost. The ICERs were also sensitive to the protective efficacy used; the use of 38.5 % protective efficacy found by Tagbor et al. [30] in the middle belt of Ghana increased the ICERs by about 1.1 times (from both provider and societal perspectives) by more than 100 % (Table 2).

### Cost and cost-effectiveness of other preventive malaria interventions

**Fig. 6:**
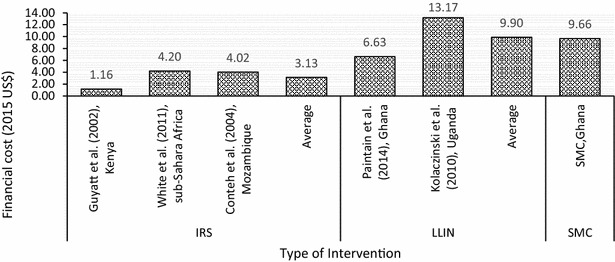
Financial cost per child by other preventive interventions

Studies have reported the financial cost per child covered by other interventions: US$9.90 (in 2015 $) in ITN campaigns in Ghana [12] and Uganda [13] and US$3.13 (2015$) in IRS programmes in Kenya [20], Mozambique [18] and SSA [21], presented in Fig. 6. However, it is quite complicated to compare different preventive interventions given the difference in the duration of efficacy of these interventions and the fact that the number of persons protected is different—for instance SMC protects only one child while ITN and IRS could protect more than one child.

**Fig. 7:**
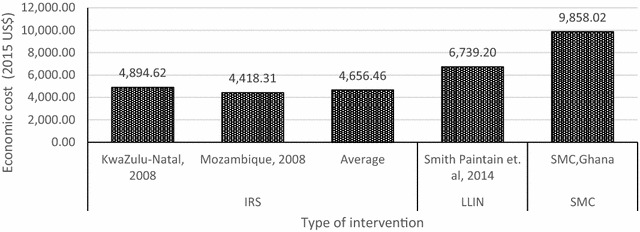
Economic cost per additional death averted

Studies have reported the economic cost (societal perspective) per death averted of other preventive malaria interventions. For example, IRS programmes in South Africa and Mozambique recorded an average ICER of US$4656.46 (in 2015$) per death averted [19]. Similarly, an ITN campaign in Ghana reported ICER of US$6739.20 per death averted [12]. Again, direct comparison of these interventions is difficult (Fig. 7).

## Discussion

The results show that the financial cost per child fully dosed (i.e., all four rounds of the survey) was US$9.66, implying that the financial cost per dosing one child per round is about US$3.25. Wa East recorded the lowest of US$4.61 and Sissala West recorded the highest financial cost per child dosed of US$26.14, although the district’s record seems to be an outlier. The finding revealed some pattern between children fully dosed and financial cost per child fully dosed. This finding implies the existence of economies of scale, where completely dosing more children leads to a fall in the average cost of dosing each child. The existence of economies of scale serves as a motivation for districts to cover as many children as possible in order to reduce their average costs. The financial cost per child fully dosed in this study compares with similar findings on ITN campaigns [12, 13]. However, lower estimates are reported for IRS [18, 20, 21]. Although the cost of medicines for SMC is expected to be higher than that of ITNs, the average cost of implementing SMC is within the range of other preventive malaria interventions. For financial cost, the main cost drivers were the dosing exercise and monitoring and supervision (both of which account for about an equal share of the total cost), and data capture.

The study further shows that the economic costs of the SMC intervention were about US$7.96 million from the societal perspective and US$2.66 million from the provider perspective, implying that if only costs from the provider perspective are considered, total costs would be less by about two-thirds. The bulk of the difference was due to valued caregiver productive losses due to participation in the intervention. The total economic cost of the intervention ranges from US$389,192.38 (DBI) to US$1175,496.28 (Wa Municipal) for societal costs and US$133,891.17 (DBI) to US$347,268.45 (Wa East) for provider perspective costs. The differences in these costs across districts seem to stem from the land area covered as well as the number of children, volunteers and caregivers in each district. Districts covering smaller land area and smaller population of number of children, caregivers and volunteers tend to record lower economic costs.

For economic costs (societal perspective), the main cost drivers were the dosing exercise (which accounts for more than half of the total), social mobilization and monitoring and supervision. These three activities used significant volunteer and caregiver time, confirming the important role that caregivers play in any community-based intervention, as these people are not remunerated. This is also confirmed by the result that indirect costs accounted for about three-quarters (i.e., 74 %) of the total economic cost from the societal perspective but only a quarter of the total economic cost from the provider perspective. While arguing that some compensation for caregivers may seem uneconomical, it is important to acknowledge their role and, perhaps, implement interventions by targeting their availability. For instance, the dosing exercise could be held in the evenings, when caregivers do not have to sacrifice productive time to be involved in the activity, which could significantly reduce the productivity loses. The dosing exercise, social mobilization, and monitoring and supervision also benefitted from donations from other agencies. For instance, in most districts, social mobilization and monitoring and supervision benefited from use of vehicles and equipment from other agencies within the districts but outside the health system. These were accounted for in estimating societal costs.

This study has estimated the additional number of under-five malaria cases averted in the region to be 24,881. This represents a 48 % reduction in malaria caseload due to SMC. Given the burden that malaria places on this vulnerable group, the SMC intervention has led to considerable reduction in the burden, mainly morbidity. It is difficult to compare this outcome to other interventions in Ghana, especially the ITN mass distribution campaign because that campaign used a different outcome indicator (i.e., number of children using an ITN) and targeted a different population size. However, studies have reported that ITN and IRS have protective efficacy of 50–60 % [27, 31] while SMC is reported to have protective efficacy of up to 80 % with full dose [27].

Further, this study has estimated the additional deaths averted in the region due to SMC to be 808. A study on ITN distribution and hanging in Ghana [12] reported additional number of deaths averted by region to range between 445 and 637. However, given the difference in the total population covered by the two interventions, it is difficult to directly compared these estimates.

The study finds that economic cost per child fully dosed was US$22.53 from the provider perspective and US$67.35 from the societal perspective. Further, the economic cost per additional case averted was US$107.06 from the provider perspective and US$319.96 from societal perspective. The study also finds that the SMC intervention averts one additional under-five death at cost of US$3298.36 from the provider perspective and US$9858.02 from the societal perspective. There are varied opinions regarding which thresholds to use in judging the cost-effectiveness of health interventions and these are extensively discussed in Culyer [32]. However, in line with other studies [24], we use the WHO-recommended thresholds—that health interventions are highly cost-effective if ICER is less than GDP per capita and cost-effective if ICER falls within one to three times GDP per capita. The Ghana Statistical Service [33] estimates Ghana’s 2015 GDP per capita to be US$1339.00. Thus, SMC intervention is highly cost-effective in terms of economic cost per additional case averted at the districts and regional levels irrespective of the perspective used in analysing cost.

In terms of cost per additional deaths averted, the SMC intervention is cost-effective at regional level and at districts (except for Sissala West and Wa Municipal) from the provider perspective. However, the intervention is not cost-effective from the societal perspective as the ICERs were higher than US$4017.00. Although the societal perspective is important for assessing cost-effectiveness of interventions that use substantial community resources, considering that many time-related community resources, especially from caregivers who hold the health of their children above their time, tend to be community contributions for which remunerations are not expected (although they come with opportunity costs), it may not be out-of-place to consider the provider perspective in deciding the worth of a programme.

The ICERs were sensitive to the choice of mortality rate used. Understandably, it could be argued that the mortality rate of 11.36 deaths per 1000 population used to estimate the ICERs in this study is an old estimate. However, updating that estimate requires collecting large, context-specific data, which can be a complex exercise. Besides, that mortality rate was the basis for estimating malaria mortality presented in some World Malaria Reports (e.g., in 2008) and other studies [27]. Further, although a study in Ghana found protective efficacy of 38.5 %, the Ashanti Region where that study was conducted has different climatic conditions compared to the Upper West Region. Consequently, the rate of 80 % protective efficacy is best for this study given that the Upper West Region is among the Regions with highest burden of malaria in Ghana (37.8 %) compared with 16.6 % prevalence in the Ashanti Region.

The cost per additional death averted (from societal perspective) for this study is higher compared to those of other studies in evaluating other preventive malaria interventions. However, the cost per additional death averted from provider perspective and economic cost per additional case averted estimated from this study are comparable to those of the other studies.

The study team observed some constraints to the SMC implementation approach used. For example, the implementation of the exercise during the farming season makes it difficult for caregivers to fully participate and this could affect the outcome of the exercise. However, as stated elsewhere in this paper, implementation of the SMC intervention is recommended for the season. Future dosing exercises may be scheduled to allow volunteers to visit households in the evening during which time caregivers would have returned from the farm. This could improve their participation and enhance outcomes, but also reduce their productive losses to caregivers. Further, the use of AMOFAN tablets, which volunteers find difficult to crash, posed some challenges to the administration of the medicine. Future exercises could consider using dispersible medicine.

Some limitations of the study are worthy to note. First, the estimation of effects of the intervention had to rely on parameters from the literature (although these were combined with prevalence obtained locally) because data on number of cases and deaths averted were not available. Thus, the rates used may influence the true impact of SMC in Ghana. Second, it was assumed that the effects of the intervention were observed only in children who received the complete course (i.e., four course/fully dosed). Thus, the standard 80 % protective efficacy was applied to the number of children who received all four courses. However, it has been shown [27] that even in children who receive three courses, protective efficacy could be up to 65 %. These effects could not be added to the model as the data available at the study site did not distinguish between the number of children who received three doses but could not receive four. Thus, the ICERs would be different from the estimated results if the 65 % protective efficacy had been calculated for those who received three doses and added to the 80 % efficacy of four doses.

## Conclusions

The financial cost per the SMC intervention delivered to a child under-five was US$9.66. Due to economies of scale, it is possible to deliver the intervention to more children at less than US$9.66. This represents a good use of society’s money and favours scaling-up of the intervention. The economic cost per additional case averted of US$107.06 and US$319.96 also represents value-for-money (i.e., cost-effective). The findings of this study show that the SMC intervention is economically beneficial in reducing morbidity in children under-5 years and presents a viable approach to improving under-five health in Ghana. Therefore, the SMC intervention is recommended for scaling-up as it represents a good use of economic resources from the provider perspective.

## Abbreviations

ADR: adverse drug reaction
AQ: amodiaquine
DFID: Department for International Development
DHMT: District Health Management Team
ICER: incremental cost effectiveness ratio
IPTc: intermittent preventive treatment in children
IPTp: intermittent preventive treatment in pregnancy
IRS: indoor residual spraying
ITN: insecticide-treated net
LLIN: long-lasting insecticide-treated net
NMCP: National Malaria Control Programme
RHMT: Regional Health Management Team
SMC: seasonal malaria chemoprevention
SP: sulfadoxine–pyrimethamine
SSA: Sub-Saharan Africa
WHO: World Health Organization
